# The Welfare of Cattle, Sheep, Goats and Pigs from the Perspective of Traumatic Injuries Detected at Slaughterhouse Postmortem Inspection

**DOI:** 10.3390/ani11051406

**Published:** 2021-05-14

**Authors:** Lenka Valkova, Vladimir Vecerek, Eva Voslarova, Michal Kaluza, Daniela Takacova

**Affiliations:** 1 Department of Animal Protection and Welfare and Veterinary Public Health, Faculty of Veterinary Hygiene and Ecology, University of Veterinary Sciences Brno, 612 42 Brno, Czech Republic; H20334@vfu.cz (L.V.); vecerekv@vfu.cz (V.V.); kaluzam@vfu.cz (M.K.); 2 Department of Public Veterinary Medicine and Animal Welfare, University of Veterinary Medicine and Pharmacy in Kosice, 041 81 Kosice, Slovakia; Daniela.Takacova@uvlf.sk

**Keywords:** cattle, sheep, goat, pig, limb trauma, trunk trauma, postmortem examination, welfare

## Abstract

**Simple Summary:**

The occurrence of traumatic injuries was assessed in cattle, sheep, goats and pigs reared and slaughtered in the Czech Republic. For the purposes of the study, the results of veterinary postmortem examinations at slaughterhouses in the period from 2010 to 2019 were analyzed. In the studied animal species, findings of traumatic lesions were detected at low frequency. The low frequency of traumatic lesions is favorable from the perspective of the welfare of slaughtered animals. In terms of further improvements to animal welfare, it would be desirable to focus on the prevention of trauma in cattle in particular, in which findings of trauma were more frequent than in the other species studied. The category most affected by trauma both to the limbs and body was cows.

**Abstract:**

The welfare of cattle, pigs, sheep and goats was assessed by measuring trauma detected during veterinary postmortem inspection at slaughterhouses. The subject of this evaluation were all bovine, porcine, ovine and caprine animals slaughtered at Czech slaughterhouses in the monitored period, i.e., a total of 1,136,754 cows, 257,912 heifers, 1,015,541 bulls, 104,459 calves, 586,245 sows, 25,027,303 finisher pigs, 123,191 piglets, 22,815 ewes, 114,264 lambs, 1348 does and 5778 kids. The data on the numbers of traumatic findings were obtained retrospectively from a national veterinary database collecting data from slaughterhouse postmortem examinations. The results showed that findings of trauma were observed at a low frequency in the studied species. Injuries were detected most frequently in cows (1.71%). In contrast, no findings associated with the presence of trauma were recorded in does and kids. From the viewpoint of trauma localization, findings on the limbs were more frequent than findings on the body (*p* < 0.01). The only exceptions to this were lambs, does and kids, for which there was no statistically significant difference between findings on the limbs and the body (*p* = 1.00). The results show that housing system (bedding, the presence of slats, floor hardness), transport of animals to the slaughterhouse (moving animals to the vehicle, loading ramps, floors in transport vehicles and the transport of animals itself) and design of the slaughterhouse (unloading ramps, passageways and slaughterhouse floors) have a greater impact on the limbs than the bodies of animals in the majority of species. A difference was also demonstrated in the occurrence of findings of trauma in the limbs and body (*p* < 0.01) between culled adult animals and fattened animals, namely in cattle and pigs. A difference (*p* < 0.01) between ewes and lambs was found only in the occurrence of traumatic injury to the limbs. The results showed that fattened animals are affected by the risk of trauma to a lesser extent than both culled adult animals and young animals. Statistically significant differences (*p* < 0.01) were also found between the studied species and categories of animals. The category most affected from the viewpoint of injury both to the limbs and body was cows. In contrast to cows that are typically reared indoors, the low frequency of traumatic findings was found in small ruminants and in bulls, i.e., animals typically reared outdoors. Assumedly, access to pasture may be beneficial considering the risk of traumatic injury.

## 1. Introduction

The welfare of livestock animals is an issue of interest to consumers [1]. The public is concerned about conditions in the rearing, transport and slaughter of livestock animals [2]. Knowledge of animal physiology, behavioral patterns and needs is essential to ensure the corresponding welfare. It is necessary to limit the risk of traumatic injury to prevent animals from pain and suffering [3]. The pain resulting from injury may be acute or chronic. For the animal, it is an unpleasant emotional experience that indisputably has a negative impact on the welfare of the individual [1]. Injury also leads to the animal’s fear of further possible traumatic injury, which would be a source of additional pain [4].

Presence of injuries is a significant indicator of impaired animal welfare [5,6]. Trauma can be assessed in live animals at the slaughterhouse, though a more-detailed evaluation can be made after slaughter during a postmortem veterinary examination [7]. Veterinary examination at the slaughterhouse is an important tool for evaluating both the state of health and the welfare of slaughtered animals [8,9]. Data on findings of trauma can be subsequently used to assess indirectly whether animals were exposed to pain as a result of injury before slaughter [10].

Injuries to livestock animals can occur on farms, during transport and at the slaughterhouse [11,12,13]. The environment on farms may itself be a cause of traumatic injury. Unsuitable floors, damaged facilities, the construction of stalls and the inadequate maintenance of rearing establishments may pose a risk of injury. Sharp objects on which animals can easily injure themselves pose a particular risk on farms [14]. Injuries may also occur as a result of the unsuitable grouping of animals on farms [15]. Social mixing is considered a significant stressor undermining animal welfare [11]. The interaction of animals of various age categories, weight or origin may be a cause of stress and mutual conflict that may lead to injuries on the farm, during transport or during lairage before slaughter.

Animals may also be injured during transport [11] or due to improper handling during loading and unloading [16]. The frequency of injury is also influenced by the behavior of the animals. Minka and Ayo [17] found that a longer loading period and the behavioral activity of the animals were associated with a higher frequency of injury. Physical contact leads to interaction and a risk of fighting between animals [18]. Other significant factors affecting the occurrence of injuries are stocking density, style of driving and duration of the journey [19,20,21]. The stocking density should be neither too high nor too low, since even a larger space may lead to injuries among animals as a consequence of trampling, aggressive clashes or falls resulting from sudden acceleration or deceleration of the vehicle [22]. Bethancourt-Garcia et al. [23] reported a greater risk of injury for animals transported in larger trucks or when load density exceeded 431 kg/m^2^. Animals may also be injured as a result of the poor condition of the vehicle or the roadway [21]. A smooth ride and the correct handling of animals, in contrast, reduces the risk of injuries occurring to animals during transport [24]. Traumatic lesions resulting from transport were documented by many studies [20,25,26]. Dalla Costa et al. [27] found that loading, transport, unloading and lairage at the slaughterhouse doubled the original number of skin lesions in pigs (from 29% to 62%). Minka and Ayo [17], who monitored the welfare of cattle during loading, transport and unloading, found bruises and lacerations on the chest wall and abdominal wall to be the most common injuries occurring during transport.

Injuries also occur at the slaughterhouse, as was demonstrated by various studies in cattle, sheep and pigs [28,29,30]. Inappropriate handling, along with excessive use of electric prods and an unfamiliar environment are factors that lead to considerable stress in animals at slaughterhouses. The stress experienced by the animals has a negative impact on their welfare [12,31]. Stress may elicit a defensive reaction or other excessive reaction from animals, which increases the risk of their injury under the given conditions [32]. According to Strappini et al. [10], of the five preslaughter stages (loading, transport, unloading, lairage and time in the stunning box), most bruises are the result of circumstances at the slaughterhouse. Ramps pose a problem to pigs [33]; they must therefore be constructed so as to ensure sufficient grip and prevent the animals from slipping. Narrow passageways and the gangway leading to the stunning box or other space where stunning occurs also pose a risk of injury for animals. The frequency of bruises at slaughterhouses is also increased by the length of lairage time. An increased number of bruises during a longer period of lairage was reported by a number of studies in various species (in cattle [30,34]; in sheep [28]; in pigs [29]).

Injuries occurring to livestock animals may be of varying extent and severity. They may occur on the body or the limbs. In addition to surface skin lesions, injuries detected in livestock animals may also include dislocations and fractures. Infection, which may lead to the secondary formation of abscesses, is closely associated with the presence of injuries [35]. Bruises are a characteristic finding of trauma that may be recorded during veterinary examinations. During postmortem inspection at the slaughterhouse, bruises appear as changes in color evident after skinning [36]. The age and cause of such lesions can be judged from an assessment of appearance, color and location [37]. Bruises may occur at any time during rearing and transport or immediately before slaughter [10,30]. Bruises are the result of a direct contact with structures of the facility [38]. The presence of bruised tissue after slaughter is an indicator of an unsatisfactory animal environment [39], inappropriate handling and pain experienced by the animal [38]. Bruises are manifested by the accumulation of blood in the muscles and other tissues as the result of the rupturing of blood vessels [36]. Their presence in cattle was reported worldwide, e.g., in Great Britain [40], the USA [41], Mexico [42], Italy [36], Uruguay [24] and Colombia [34]. Bruises were also reported in sheep [28,43]. In pigs, various degrees of skin lesions resulting from biting were also reported, in addition to bruises [44]. Furthermore, limb injuries were also detected [45].

The aim of this study was to assess the welfare in cattle, sheep, goats and pigs based on the findings of trauma detected after slaughter at the slaughterhouse. The occurrence of traumatic injuries was also monitored in the studied animal species from the viewpoint of their localization (the limbs and body).

## 2. Materials and Methods

The occurrence of traumatic injuries was assessed in cattle, sheep, goats and pigs reared and slaughtered in the Czech Republic retrospectively on the basis of an analysis of the results of veterinary postmortem examinations at slaughterhouses in the period from 2010 to 2019. The subject of this evaluation were all bovine, porcine, ovine and caprine animals slaughtered at Czech slaughterhouses in the monitored period, i.e., a total of 1,136,754 cows, 257,912 heifers, 1,015,541 bulls, 104,459 calves, 586,245 sows, 25,027,303 finisher pigs, 123,191 piglets, 22,815 ewes, 114,264 lambs, 1348 does and 5778 kids slaughtered at 226 cattle slaughterhouses, 209 pig slaughterhouses, 146 sheep slaughterhouses and 81 goat slaughterhouses. Animals that died or were killed on farms (emergency killing) because they had been considered unfit for transport to the slaughterhouse (according to the Council Regulation (EC) No 1/2005 [46]) were not included in the analysis in our study.

For the purposes of this study, the categories of animals in individual species were further assigned to the groups of culled adult animals (cows, sows, ewes, does; 1,747,162 animals in total), fattened animals (heifers, bulls, finisher pigs, lambs and kids; 26,420,798 animals in total) and young animals culled from the herd (calves and piglets; 227,650 animals in total).

The data on the numbers of traumatic findings were obtained from a veterinary database collecting data from the slaughterhouse postmortem examinations. At the slaughterhouses, the assessment of traumatic injuries in animals was performed by official veterinary surgeons designated by the State Veterinary Administration. The classification of findings was based on the methodology for postmortem examinations of animals at slaughterhouses. All animals slaughtered at slaughterhouses (their carcasses) are inspected by certified veterinary inspectors who received a uniform training and certification of competence to perform this kind of veterinary slaughterhouse inspections. The legal requirements and specification of veterinary postmortem examination are laid down by European Union legislation [47].

During postmortem examinations, veterinary inspectors differentiated between injury to the body and injury to the limbs. Open wounds at various stages of healing, hematoma in the hypodermis and muscles, bruises, dislocations, fractures (open and closed) and other changes that may arise as a consequence of the housing system used, incorrect handling and interactions between animals on the farm, during transport or during lairage before slaughter were included among findings of trauma. However, veterinary inspectors did not aim to classify the cause of the antemortem injuries. They only distinguished injuries that occurred ante- and postmortem on the basis of their appearance (observation of biological processes related to tissue regeneration, presence of clotting, swelling, inflammation, scarring…). Postmortem injuries (technology-related damage following stunning) were not included among findings of trauma analyzed in our study.

The occurrence of trauma was further evaluated according to injury localization. The frequency of traumatic injuries in the limbs and on the body was compared.

Differences in the occurrence of traumatic injury to the limbs and body between adult animals and fattened animals were further studied in individual species (cattle, pigs, sheep and goats).

The results were assessed statistically with the program Unistat 6.5 for Excel (Unistat Ltd., London, UK). Statistical comparisons between the frequencies of the categorical variables of interest were performed using the Chi-square test (with Yates correction) within the 2 × 2 contingency table procedure [48]. When the frequencies in the contingency table were lower than five, a Fisher exact test was used instead of the Chi-square test [48].

## 3. Results

The frequency of occurrence of findings of trauma in the studied categories of cattle, sheep, goats and pigs is given in Table 1. Significant differences in the frequency of traumatic injuries were found among the monitored species (Table 2). The results show that traumatic injuries were detected most frequently in cows (1.71%), while no traumatic injuries were found in does or kids.

**Figure 1:**
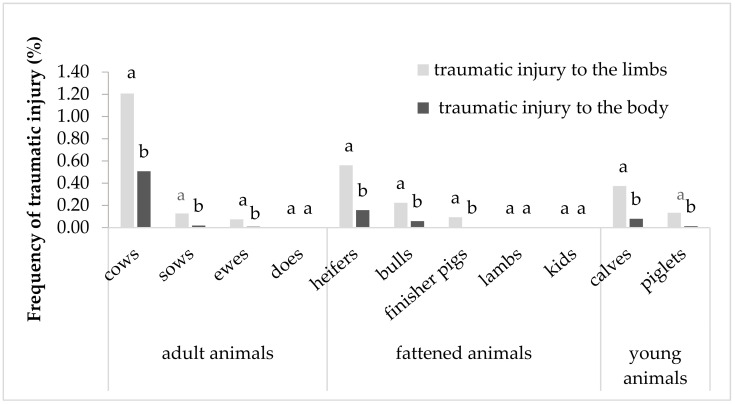
Comparison of the frequency of traumatic injury to the limbs and body in slaughter animals. ^a,b^ different superscripts by the number of findings on the limbs and body within the same species and category of animals indicate a statistically significant difference (*p* < 0.01).

A comparison of the frequency of findings of trauma between the limbs and the body in individual species and categories of cattle, sheep, goats and pigs is given in Figure 1. The results show that traumatic injury to the limbs was recorded more frequently (*p* < 0.01) than traumatic injury to the body in the majority of the studied species and categories of animals. The only exceptions to this were lambs, does and kids. No findings associated with traumatic injury to the limbs or body were recorded in does or kids. In lambs, two (0.002%) findings on the limbs and three (0.003%) on the body were recorded. The greatest frequency of traumatic injury was recorded in cows (limbs: 1.21%; body: 0.51%), heifers (limbs: 0.56%; body: 0.16%) and calves (limbs: 0.37%; body: 0.08%).

A comparison of the frequency of traumatic injury to the limbs and the body in adult animals and fattened animals in cattle, pigs, sheep and goats is given in Table 3. The results show a statistically significant difference (*p* < 0.01) in the number of traumatic findings between adult animals and fattened animals in cattle (both in the limbs and body), i.e., between cows and heifers and also between cows and bulls. A statistically significant difference (*p* < 0.01) was also found between adult animals and fattened animals in pigs (both in the limbs and the body), i.e., between sows and finisher pigs. In sheep, a statistically significant difference between ewes and lambs was found only in the occurrence of traumatic injury to the limbs (*p* < 0.01). No traumatic injury to either the limbs or the body was recorded in slaughtered does and kids.

A comparison of the frequency of traumatic injury to the limbs and body in individual species in culled adult animals, fattened animals and young animals culled from the herd is given in Table 4.

The results show that among culled adult animals, traumatic injury to the limbs and body most affected cows, with statistically significantly fewer (*p* > 0.05) findings of trauma recorded in sows, ewes and does. Among fattened animals, the largest number of findings of trauma in the limbs and body was recorded in heifers and then in bulls. In young animals culled from the herd, significantly more (*p* > 0.01) findings of trauma in the limbs and body were recorded in calves than in piglets.

## 4. Discussion

A high number of traumatic lesions is detected in livestock at the slaughterhouse, particularly in developing countries [11,49]. Bueno et al. [50], who studied the causes of condemnation in pigs at slaughterhouses in Brazil, reported that findings of trauma leading to condemnation included abscesses (28.9%), fractures and hematomas (26.7%), and bruises (0.28%). In Europe (as documented by the previous studies conducted in the Czech Republic), findings of trauma are more frequent in animals reared and transported in cages, i.e., poultry [51,52] and rabbits [53], than in animals reared and transported unrestrained, i.e., cattle [54,55] and pigs [35,56]. The results of our study also show that findings of trauma were recorded in cattle, sheep, goats and pigs only at low frequency, not exceeding 1% in any of the species monitored in this study with the exception of cows, in which total findings of trauma amounted to 1.71%. No findings associated with the presence of trauma were recorded at the slaughterhouse in does and kids. The low frequency of traumatic lesions is favorable from the perspective of the welfare of slaughtered animals; it may indicate satisfactory conditions on farms, during transport and during lairage at slaughterhouses.

### 4.1. Localization of Traumatic Lesions in Cattle, Sheep, Goats and Pigs

An unsuitable environment leads to diverse injuries in animals, affecting various parts of the body. Any lesions on the limbs or body of livestock animals have a negative effect on their welfare [37,45]. The results of our study show that traumatic lesions of the limbs occur significantly more often than traumatic lesions of the body in all categories of animals (adult animals, fattened animals, young animals). Similar findings have also been reported in other studies evaluating findings of trauma on farms or during veterinary examinations in cattle and pigs. Weary and Taszkun [57] found that injuries to cattle generally appear on various protuberances, usually the joints; namely the tarsal joint injuries being very frequent. KilBride et al. [45], who studied the prevalence of lesions on the limbs and body of lactating sows in England, found the prevalence of lesions on the limbs (93%) to be considerably higher than on the body (20%). According to Herskin et al. [58], ulcers on the limbs in the shoulder joint area are common in sows. Bonde [59] recorded decubitus ulcers in 17% of lactating sows. Quinn et al. [60], who studied the presence of limb lesions in unweaned piglets, reported frequent sole bruises (61.5%), abrasions on the limbs (55.7%) and sole erosion (34.1%). Harley et al. [61], who analyzed findings at pig slaughterhouses, revealed that 16% of slaughtered pigs were affected by severe bruises and 44% had bursitis in the hind limb. A greater occurrence of pathological findings in the limbs than on the body in pigs was also reported by Vecerek et al. [35] in all pig categories (sows, finisher pigs and piglets). In contrast, Petersen et al. [62], who monitored the prevalence of clinical symptoms in pigs in the final phase of fattening in Denmark, found a predominance of injuries to the body: ear necrosis (4.44%) and bite wounds to the tail (1.26%) and flanks (0.52%).

### 4.2. The Occurrence of Traumatic Lesions in Culled Adult Animals and Fattened Animals

The results of our study show that there is a significant difference in the occurrence of traumatic lesions between culled adult animals and fattened animals in most of the monitored species. This difference was found in lesions both on the limbs and body in cattle and pigs, and also in sheep even if only in the limbs. The only exception was goats, in which no findings of trauma were recorded. The number of does and kids transported to slaughterhouses was, however, many times lower in comparison with the other categories and this may have influenced the results.

The duration of exposure to factors responsible for trauma may be a cause of the differences between culled adult animals and fattened animals. The period adult animals stay on the farm is longer than that of fattened animals, which are sent to the slaughterhouse before reaching adulthood, for which reason the probability of traumatic injury may be greater in adult animals, as is shown by the results of our study. Strappini et al. [38], who studied the prevalence of bruises in cattle at selected slaughterhouses in Chile, also detected bruises more often in older categories of animals. Older animals are usually larger and heavier and their size may be a confounding factor.

In addition to age and size, other factors must also be taken into consideration during assessment of the degree of risk of injury, with sex, castration, category of animal and body fat cover also having an influence on the possible occurrence of trauma [63,64,65]. Differences in the amount of deposited fat may be a cause of more frequent bruises in female cattle [66]. Strappini et al. [38] reported a lower occurrence of bruises in animals that do not resist handling. Furthermore, they reported cows to be more susceptible to bruising compared to young animals, heifers or castrated bulls. Bethancourt-Garcia et al. [23] found that cows had a markedly higher prevalence of bruises (79.13%) than bulls (20.87%). This is in agreement with the results of our study, in which the presence of findings of trauma was 6 times higher in cows and 2.5 times higher in heifers than it was in bulls. Weeks et al. [40] obtained similar results and reported bulls to have the lowest number of traumatic injuries during veterinary examination. They found a larger number of injuries in heifers and steers and recorded the largest number of injuries in cows. Other studies also draw attention to the greater sensitivity of cows to bruises in comparison with fattened cattle [41,64]. A certain role may also be played by breed, which influences the behavior of the animals. According to Lee et al. [67], Holstein cattle show a greater prevalence of bruises among cattle.

A lower level of traumatic findings among fattened animals was recorded in pigs and sheep in our study. The frequency of trauma was 1.5 times higher in sows than in finisher pigs (0.15% as opposed to 0.10%). In sheep, findings of trauma were 18 times more frequent in adult sheep than in lambs (0.083% as opposed to 0.004%). In contrast, Cockram and Lee [28], who studied the occurrence of bruises on sheep carcasses, found a greater frequency of bruises in lambs than in ewes. They also found that the majority of bruises happened before arrival at the slaughterhouse.

The factors that may influence the presence of traumatic findings also include the animals’ state of health, condition and healing ability, which is greater in fattened (younger) animals than in adult (older) animals. Adult animals are usually culled from the herd and sent to slaughter due to reproductive problems, reduced health and age [68,69,70], while fattened animals are sent to slaughter in their prime.

However, culling is also performed in groups of young animals. Piglets and calves are usually not slaughtered in the Czech Republic unless they are culled and sent to a slaughterhouse due to deficiencies in their physical and/or health condition [35,54]. Correspondingly, our results show that the frequency of traumatic findings is greater not only in culled adult animals but also in culled young animals compared to fattened animals. A frequency of findings of trauma 1.6 times higher was recorded in calves than in bulls (0.45% as opposed to 0.28%). Similarly, the frequency of trauma was 1.5 times higher in piglets than in finisher pigs (0.15% as opposed to 0.10%).

### 4.3. The Occurrence of Traumatic Injuries to the Limbs in Individual Animal Species

The results of our study show a difference in the occurrence of traumatic injuries to the limbs between individual animal species. In all groups (culled adult animals, fattened animals, young culled from the herd), the largest number of findings was made in cattle, followed by pigs and sheep, with no findings being made in goats. According to our results, the discrepancy between the welfare requirements and housing and transporting conditions (considering the risks of limb injuries) differs between individual animal species, with this discrepancy being most pronounced in cattle, followed by pigs, and smallest in sheep and goats.

A nonslippery soft and dry earthen floor is desirable to keep limbs in good condition in cattle. The greater occurrence of traumatic findings in the limbs is probably associated with contemporary farming systems (particularly in dairy cows and heifers). Cattle face a particular risk of limb injuries on intensive farms [71]. Rutherford et al. [72] recorded a greater occurrence of limb injuries on intensive farms (49.1%) than on organic farms (37.2%). Extensive farming conditions may be favorable from the viewpoint of limb health. Lee et al. [67] found injuries to cattle to be more frequent in the Holstein breed than in beef breeds. The results of our study also demonstrated that dairy cows are affected by limb injuries more than other categories of cattle. The frequency of traumatic limb injuries was six times higher in cows than in bulls (1.21% as opposed to 0.22%). Heifers were less affected by limb injuries (0.56%), which is evidently due to the fact that these are younger animals that are generally also kept on pasture, no matter whether they are dairy or beef. Fat cattle are reared primarily on pasture in the Czech Republic [69], where they may be exposed to the risk of limb injuries if kept on steep unconsolidated areas or in significantly waterlogged paddocks adjoining sheds. It is, however, clear from our results that the risk of injury is lower than for indoor-housed animals, evidence of which is provided by the lower level of findings of trauma in the limbs of heifers and bulls. In cattle housed indoors, factors affecting the risk of injury to the limbs include bedding material [57,73,74,75,76,77], quality and type of floors [1,57,73,78,79], the total pen space per cow [57], the length of the grazing period during the year [80] and the number of lactations [57].

In pigs, the type of floors also has a major effect on their limb health. The greater prevalence of limb lesions resulting from housing on a hard floor was reported in piglets [60], in finisher pigs [35] and in sows [35,58,81,82]. Floors could be a serious problem for all categories of pigs in intensive farming but they are more frequently used in sows and piglets. Correspondingly, the results of our study show the frequency of limb injuries to be 1.5 times higher in sows and piglets than in finisher pigs. From the perspective of the occurrence of limb injuries, outdoor housing proved to be a better option compared to indoor housing [45]. Outdoor housing for pigs is, however, rare in the Czech Republic and is found only on small farms and organic farms. In intensive farming, contact with the outside environment is precluded for biosecurity reasons. According to the results of our study, the risk of limb injuries is rare in sheep in comparison with cattle and pigs, as indicated by a significantly lower incidence of findings of trauma on the limbs in sheep. It is possible that the herd reflex in sheep reduces the negative effect of slippery floors, with the animals leaning on one other when sheep are moved, thereby restricting the frequency of occurrence of traumatic limb imbalance and falls. The results also showed that limb injuries were recorded only twice in lambs during the entire monitored period, and not at all in does and kids. The low level of limb injuries in small ruminants is probably conditioned by the farming system, the biology and behavior of the animals, and the method of handling. Lambs and goats are transported with a greater degree of consideration in view of their greater nervous sensitivity, with the speed of movement during loading and unloading corresponding to the biology of the animals, for which reason significant traumatic injuries do not occur. Positive human–animal interaction reduces stress in animals, contributes towards their better welfare and may also reduce the risk of injury to the animals [83,84]. The farming of small ruminants in extensive conditions and, particularly in the case of goats, on small farms predominates in the Czech Republic. The threat of injury to these animals comes primarily from encounters with predators [85]. Such cases generally lead to death or the necessity of euthanasia on the farm, however. The risk of injuries that could be evident at the slaughterhouse arises primarily during transport. In the Czech Republic, however, small ruminants are generally transported to local slaughterhouses in small-capacity vehicles. A smaller volume of transported animals, their herd instinct, environmental conditions and handling conditions are factors that may significantly reduce the risk of injury, and not merely to the limbs, in small ruminants.

### 4.4. The Occurrence of Traumatic Injuries to the Body in Individual Animal Species

Traumatic injuries to the body were recorded to a lesser extent than traumatic injuries to the limbs in most species and categories. The low frequency of findings led in certain cases to the disappearance of differences between individual species in culled adult animals and fattened animals. The findings of trauma on the body were most frequent in cattle, followed by pigs and then sheep. Injuries to the body may result from non-rounded and sharp edges on railings, barriers and walls on farms, during transport or at the slaughterhouse. A risk of injury to the body is also posed by falls resulting from moving animals on unsuitable floors [14]. Satisfactory living and transport conditions can be anticipated in goats, in which no findings of trauma were made on the body.

The results of our study showed that of all the studied species and categories, cows were again the category of animals with most frequent findings of trauma on the body. However, the frequency of traumatic lesions found in heifers and calves was also significantly greater than in other monitored categories of animals. The nature of cattle may lead to more frequent driving of the animals, which may sometimes be associated with the occurrence of body injuries. Rough handling, including the use of prods, leads to the formation of bruises in cattle [13,24,36,37]. Among pigs, the category least affected by traumatic lesions on the body was finisher pigs. Body injuries were significantly more frequent in sows (6.5 times more frequent than in finisher pigs) and piglets (4 times more frequent than in finisher pigs). Injuries to the body in pigs occur most commonly as a result of an unsuitable housing system [86,87,88,89], mutual interaction (fighting) [44,87,90,91,92], unsuitable handling (e.g., excessive use of prods) and transport [90,93].

The risk of injuries to the body is slight in sheep in comparison to cattle and pigs, as can be seen from the low occurrence of traumas to the body in sheep over the ten-year period monitored in our study. The risk of injury is probably reduced in sheep by the layer and character of their coat, which dampens the impact of damage to the body caused by sharp edges or falls. Injuries may occur to sheep in the Czech Republic primarily on the farm or during transport. Foreign authors state animal markets to be the main site of the occurrence of bruising of sheep [43]. A low risk of injury to the body was also found in lambs, does and kids. Injuries to the body were recorded only three times during the entire monitored period in lambs, with none at all detected in does or kids. Presumably, in view of their greater nervous sensitivity, small ruminants are transported with a greater degree of consideration, with the speed of loading and unloading of the animals corresponding to their welfare, for which reason significant traumatic damage to the body does not occur. The risk of injury is reduced by correct handling and positive interaction with the animals on the farm, during transport and at the slaughterhouse [83,84].

### 4.5. The Sensitivity of Post-Mortem Veterinary Inspections and Their Limitations

Differences between individual countries may exist in systems for the detection of lesions [24,38], with the main reason for the variability of findings between individual countries generally being a differing approach to lesion scoring [94] and the extent of the data recorded. It would be desirable during veterinary examination to distinguish trauma arising on the farm from injuries resulting from transport or lairage at the slaughterhouse [8]. It is not always possible, however, to distinguish with any certainty the origin of such injuries during a postmortem examination [7], and the assessment of findings at the slaughterhouse may therefore be inaccurate in relation to the level of welfare on the farm and during transport [61,95]. Furthermore, animals that die or are killed on a farm are disposed of as fallen stock and thus, do not undergo postmortem slaughterhouse examination. The frequency of traumatic findings in such animals is unknown. However, in the Czech Republic, the majority of cattle, sheep, goats and pigs are slaughtered at slaughterhouses. The current extent of postmortem veterinary examinations does make it possible to point out fundamental problems on farms, during transport and at the slaughterhouse [9], particularly when they are performed over a long period of time and take in a sufficient sample of animals. The detection of traumatic injuries during veterinary examination at the slaughterhouse must be performed over a long period of time and on a sufficient number of animals for relevant conclusions to be reached. In our case, this involved a ten-year period and an extremely large number of slaughtered animals (all animals slaughtered in slaughterhouses in the Czech Republic in the monitored period). However, since the retrospective analysis of results of postmortem examinations does not provide information on origin (cause, time) of injuries detected, further research is needed to identify specific risk factors and to suggest relevant preventive and corrective measures.

## 5. Conclusions

Findings of traumatic lesions were observed at low frequency in the studied species and categories of animals. Among them, they were greatest in cattle, followed by pigs and then sheep. No traumatic lesions at all were found in goats. The low frequency of traumatic lesions is favorable from the perspective of the welfare of slaughtered animals. In terms of further improvements to animal welfare, it would be desirable to focus on the prevention of trauma in cattle in particular, in which findings of trauma were more frequent than in the other species studied in the group of culled adult animals (cows), the group of fattened animals (heifers, bulls) and the group of young animals culled from the herd (calves). In order to achieve this, further research should focus on identification of factors reducing the risk of injury, namely in cattle.

## Figures and Tables

**Table 1:** Frequency of traumatic injuries in monitored animal species.

	Cattle				Pigs			Sheep		Goats	
	Cows	Heifers	Bulls	Calves	Sows	Finisher Pigs	Piglets	Ewes	Lambs	Does	Kids
Number of slaughtered animals	1,136,754	257,912	1,015,541	104,459	586,245	25,027,303	123,191	22,815	114,264	1348	5778
Number of traumatic injuries	19,484	1853	2865	474	860	24,218	179	19	5	0	0
Frequency of traumatic injuries (%)	1.714 ^a^	0.718 ^b^	0.282 ^d^	0.454 ^c^	0.147 ^e^	0.097 ^g^	0.145 ^e,f^	0.083 ^f,g,h^	0.004 ^e,i^	0.000 ^d,f,g,h,i^	0.000 ^h,i^

^a–j^ percentages with different superscripts differ (*p* < 0.05).

**Table 2:** The pairwise comparisons (*p*-value) of frequency of traumatic injuries in monitored animal species.

Species/Category										
	cows									
heifers	0.000	heifers								
bulls	0.000	0.000	bulls							
calves	0.000	0.000	0.000	calves						
sows	0.000	0.000	0.000	0.000	sows					
finisher pigs	0.000	0.000	0.000	0.000	0.000	finisher pigs				
piglets	0.000	0.000	0.000	0.000	0.940	0.000	piglets			
ewes	0.000	0.000	0.000	0.000	0.017	0.583	0.025	ewes		
lambs	0.000	0.000	0.000	0.000	0.000	0.000	0.000	0.000	lambs	
does	0.000	0.003	0.090	0.023	0.293	0.481	0.299	0.576	0.943	does
kids	0.000	0.000	0.000	0.000	0.006	0.031	0.007	0.056	0.781	1.000

**Table 3:** Comparison of the frequency of traumatic injury to the limbs and body in adult animals and fattened animals in cattle, pigs, sheep and goats.

Species	Category	Number of Slaughtered Animals	Traumatic Injury to the Limbs (%)	Traumatic Injury to the Body (%)
cattle	cows	1,136,754	1.207 ^a^	0.507 ^a^
	heifers	257,912	0.561 ^b^	0.157 ^b^
	bulls	1,015,541	0.223 ^c^	0.058 ^c^
pigs	sows	586,245	0.128 ^a^	0.019 ^a^
	finisher pigs	25,027,303	0.094 ^b^	0.003 ^b^
sheep	ewes	22,815	0.075 ^a^	0.009 ^a^
	lambs	114,264	0.002 ^b^	0.003 ^a^
goats	does	1348	0.000 ^a^	0.000 ^a^
	kids	5778	0.000 ^a^	0.000 ^a^

^a–c^ percentages with different superscripts in the individual categories of animals within the same species differ (*p* < 0.01).

**Table 4:** Comparison of the frequency of traumatic injury to the limbs and body in individual species in adult animals, fattened animals and young animals.

Category	Species	Number of Slaughtered Animals	Traumatic Injury to the Limbs (%)	Traumatic Injury to the Body (%)
adult animals	cows	1,136,754	1.207 ^a^	0.507 ^a^
	sows	586,245	0.128 ^b^	0.019 ^b^
	ewes	22,815	0.075 ^c^	0.009 ^b^
	does	1348	0.000 ^b,c^	0.000 ^b^
fattened animals	heifers	257,912	0.561 ^a^	0.157 ^a^
	bulls	1,015,541	0.223 ^b^	0.059 ^b^
	finisher pigs	25,027,303	0.094 ^c^	0.003 ^c^
	lambs	114,264	0.002 ^d^	0.003 ^c^
	kids	5778	0.000 ^d^	0.000 ^b,c^
young animals	calves	104,459	0.374 ^a^	0.080 ^a^
	piglets	123,191	0.133 ^b^	0.012 ^b^

^a–d^ percentages with different superscripts for individual species of animals within the same group (adult animals, fattened animals, young animals) differ (*p* < 0.05).

## Data Availability

Data for analysis was obtained from the information system of the State Veterinary Administration of the Czech Republic. The datasets generated and analyzed during the current study are available from the corresponding author on reasonable request.
